# Availability of over-the-counter antibiotics in Guatemalan corner stores

**DOI:** 10.1371/journal.pone.0239873

**Published:** 2020-09-25

**Authors:** Purificación Moreno, Alejandro Cerón, Karen Sosa, Marinees Morales, Laura M. Grajeda, Maria Renee Lopez, John P. McCraken, Celia Cordón-Rosales, Guy H. Palmer, Douglas R. Call, Brooke M. Ramay

**Affiliations:** 1 Center for Health Studies, Universidad del Valle de Guatemala, Guatemala City, Guatemala; 2 Department of Pharmaceutical Chemistry, Universidad del Valle de Guatemala, Guatemala City, Guatemala; 3 Department of Anthropology, University of Denver, Denver, CO, United States of America; 4 Paul G. Allen School for Global Animal Health, Washington State University, Pullman, WA, United States of America; College of Pharmacy & Health Sciences, UNITED STATES

## Abstract

Widespread availability of antibiotics without prescription potentially facilitates overuse and contributes to selection pressure for antimicrobial resistant bacteria. Prior to this study, anecdotal observations in Guatemala identified corner stores as primary antibiotic dispensaries, where people purchase antibiotics without prescriptions. We carried out a cross sectional study to document the number and types of antibiotics available in corner stores, in four study areas in Guatemala. A total of 443 corner stores were surveyed, of which 295 (67%) sold antibiotics. The most commonly available antibiotics were amoxicillin, found in 246/295 (83%) stores, and tetracycline, found in 195/295 (66%) stores. Over the counter sales result from laissez-faire enforcement of antibiotic dispensing regulations in Guatemala combined with patient demand. This study serves as a baseline to document changes in the availability of antibiotics in informal establishments in light of new pharmacy regulations for antibiotic dispensing, which were adopted after this study was completed.

## Introduction

Antimicrobial resistance (AMR) is a global public health threat that challenges our ability to respond to infectious diseases [1]. Increased use of antibiotics, together with the absence or inefficiency of programs for the prevention and control of infections, inadequate AMR surveillance and insufficient regulation of antibiotics are all correlated with the spread of antimicrobial resistance [1]. From a population dynamics perspective, the more that bacteria are exposed to antibiotics, the greater the probability that new resistant strains will emerge in a population, and that resistant strains already in the population will expand in prevalence. Importantly, this “amplification” of resistant bacteria translates into higher probabilities that resistant strains will be transmitted to new hosts [2].

At the community level, one potential driver of amplification is unregulated availability of antibiotics. When this occurs, people are more likely to access and use antibiotics without any medical oversight [3]. In Guatemala, our anecdotal observations found that corner stores, although unauthorized establishments for dispensing drugs, sell antibiotics without a prescription (over-the-counter), without quality guarantees (missing package inserts, expiration dates or lot numbers) and without restrictions on dosage, number of units or pharmaceutical form [4, 5]. Although such practices are thought to be common in Guatemala, no objective studies are available to guide public health policy, education, and enforcement.

In this study, we sought to document the number and types of antibiotics available in corner stores in four study areas in Guatemala. This work was carried out between 2016 and 2019 prior to August 2019 legislation enacted by the Guatemalan legislature that requires prescriptions for sales of antibiotics. Consequently, this work represents an important baseline for future comparison after sufficient time has passed for new regulatory authority to be exercised.

## Materials and methods

A cross-sectional study was conducted to determine the availability of antibiotics in tablet or capsule form in corner stores in the four study areas that differ considerably in geographic, socio-economic, and ethnic variables as well as prevailing access to health services (Table 1) [6, 7].

**Table 1:** Characteristics of the study municipalities [6, 7].

Municipality	Population size	Area (km^2^)	Total poverty (%)^a^	Rural inhabitants (%)^b^	Main ethnicity ^c^	Public health infrastructure (number of public health facilities)^d^	Number of physicians in the public system per 10,000 inhabitants
Antigua Guatemala	46,054	78	21.9	0	Mestizo	1 hospital 1 health center 11 basic health facilities	1
Ciudad de Guatemala	923,392	996	33.3	0	Mestizo	2 hospitals 109 health centers	8.1
San Juan Ostuncalco	51,828	109	72.5	60	Maya-Mam	1 health center 5 basic health facilities	0.2
Coatepeque	105,415	400	42.8	65	Mestizo	1 hospital 1 health center 11 basic health facility	0.4

^a^ Total poverty includes extreme poverty, those who do not cover the cost of minimum food consumption, and non-extreme poverty, those who cover the cost of minimum food consumption, but not, the minimum additional cost for others basic goods and services (6,7)

^b^ People living in non-urban areas

^c^85% or more of the population

^d^ Excluding Centers of Guatemalan Institute of Social Security.

Google Earth® satellite images were used to delineate the study areas based on the observable population density within municipal borders. Borders were drawn around the most populated areas to indicate differences between commercial sectors and neighborhoods. For study sites furthest from Guatemala City, municipality maps were used to identify limits given availability and accessibility to official maps.

After delineating the study areas, a census was carried out to identify all establishments meeting the definition of “corner store,” a commercial establishment where a diversity of products are sold (e.g., household cleaning products, personal hygiene products, and basic food products) in relatively small volumes with one or more attendants who typically stand behind a counter that separates the products from the customers. Publicly available records from the municipality were obtained to identify all registered corner stores and was confirmed on the ground by verifying the geographical location and addresses. Establishments not listed in the official municipality data were also documented during the census and invited to participate during the study period. The number of stores identified was 371 for Guatemala City, 81 for Antigua Guatemala, 82 for Coatepeque, and 120 for San Juan Ostuncalco. All corner stores identified in the census were invited to participate in the study, with the exception of Guatemala City, where a high density of stores was encountered (N = 371). In this case, we used a population proportion to calculate the sample size, assuming a 50% sample proportion (in light of absence of previously published data), 5% margin of error and a 95% CI, resulting in a sample size of 189. These corner stores were assigned a random number in an excel spreadsheet using the command = RAND() and were subsequently ordered smallest to largest and visited in this order until 189 stores were enrolled.

Local field workers were hired to carry out the study and trained during a two-day workshop during which basic concepts of antibiotics (types, costs and dosing forms) census and questionnaire administration methods were covered. Then, the research team piloted the study to ensure uniformity in questionnaire administration visiting 10 corner stores in the community of Quetzaltenango, Guatemala.

Local field workers visited each establishment for study recruitment and participation by responding to a questionnaire. If the person responsible for the store at the time of the visit was interested in participating, verbal informed consent was carried out. If the store was closed or unattended at the time of the visit, local field workers returned for up to a total of three times before excluding the establishment from the study.

Questionnaires captured information on antibiotic type, manufacturer, dosage form and cost. We approached the antibiotic availability question by referring to specific antibiotics and probed respondents to show field workers all available medications (see supporting information S1 Dataset). Data was collected electronically using Google Forms® or RedCap® and sent to a secure server on the same day after completion of the questionnaire. STATA 14.0 ® software was used to carry out data cleaning and for of descriptive statistics. Research ethics committees at the Universidad del Valle de Guatemala, at the Faculty of Sciences and Humanities and at the Center for Health Studies, approved the study protocol and questionnaire.

## Results

Of the 472 corner stores approached, 443 (94%) accepted to participate and were enrolled in the study: all corner stores approached in Guatemala City and San Juan Ostuncalco participated in the study and 76% (62/82) and 95% (72/77) of corner stores participated in Antigua and Coatepeque respectively. Of the total of 443 corner stores interviewed, 295 (67%) sold antibiotics. Guatemala City (82%) and Coatepeque (81%) had the highest proportion of corner stores that stocked antibiotics (Table 2). The most commonly available antibiotics were amoxicillin, available in 246/295 (83%) stores, and tetracycline, which was found in 195/295 (66%) stores. Sulfamethoxazole-trimethoprim was found in three stores, ampicillin in two stores, ciprofloxacin in one store and azithromycin in one store. All antibiotics were sold as capsules or tablets that were packaged in individual blisters that had been manually cut-out from original manufacturer blister packs. Blisters were sold without any associated information such as expiration date, information related to the package insert, lot number or manufacturer. Amoxicillin and tetracycline were sold as 500 mg tablets or capsules. The median price per unit ranged from 0.16 and 0.19 USD (Table 2).

**Table 2:** Availability and prices of antibiotics in four municipalities of Guatemala.

**AVAILABILITY OF ANTIBIOTICS IN CORNER STORES**		
**Location**	**Number of stores surveyed**	**Stores selling antibiotics**
San Juan Ostuncalco	120	45 (38%)
Coatepeque	72	58 (81%)
Guatemala City	189	155 (82%)
Antigua Guatemala	62	37 (60%)
**Total**	**443**	**295 (67%)**
**AVAILABILITY OF AMOXICILLIN (500 mg capsules)**		
**Location**	**Amoxicillin available n = 295**	**Price per unit in USD**^**a**^ **(median / IR**^**b**^**)**
San Juan Ostuncalco	37 (82%)	0.26 (0.1; 0.3)
Coatepeque	39 (67%)	0.13 (0.1; 0.2)
Guatemala City	142 (92%)	0.20 (0.1; 0.3)
Antigua Guatemala	28 (76%)	0.26 (0.1; 0.5)
**Total**	**246 (83%)**	0.20 **(0.1; 0.3)**
**AVAILABILITY OF TETRACYCLINE (500 mg capsules)**		
**Study area**	**Tetracycline available n = 295**	**Price per unit in USD (median / IR)**
San Juan Ostuncalco	15 (33%)	0.13 (0.1; 0.3)
Coatepeque	32 (55%)	0.13 (0.1; 0.2)
Guatemala City	128 (83%)	0.16 (0.1; 0.2)
Antigua Guatemala	20 (54%)	0.20 (0.2; 0.3)
**Total**	**195 (66%)**	**0.16 (0.1; 0.2)**

^a^US dollars

^b^Interquartile Range

## Discussion

Antibiotics were widely available in the corner stores surveyed and were sold as individual capsules or tablets. These findings are similar to those found in Mexico, where the acquisition of antibiotics in corner stores and subsequent self-medication have been documented [8]. Additional studies demonstrate the availability of antibiotics in corner stores beyond Latin America, and even in the United States, where people from migrant communities living under unfavorable socio-economic conditions seek antibiotics as single-dose blister-tablets [9]. Furthermore, studies from five continents show that non-prescription antibiotic use is prevalent worldwide [3].

The high availability of antibiotics from informal establishments in Guatemala highlights the political and social attributes of medication supply and demand in this country [2, 3]. There is a high demand for antibiotics that is driven by a significant burden of disease and self-medication. This behavior may be due first, to a lack of knowledge concerning the importance of rational use of antibiotics [10], but also to commonly encountered barriers to securing appointments with physicians in the private and public health sector [7]. Medication stock-outs have also been documented in the public health sector, where medications are normally provided free of charge, and this likely drives some demand at corner stores.

Over the counter sales of antibiotics are further facilitated by the laissez-faire enforcement of antibiotic dispensing regulations in Guatemala and are re-enforced by patient demand [7]. This is particularly true for corner stores even though they are, technically, only allowed to sell over-the-counter medications used for symptomatic treatment (e.g., cold medications, aspirin, etc.). The August 2019 legislation requires prescriptions for purchase of antibiotics from pharmacies [11]. To date, however, there have been no accompanying public health educational efforts. How this law will in turn affect availability of antibiotics in informal establishments, including corner stores remains unknown. Therefore, this study serves as a baseline to document how the availability of antibiotics in informal establishments changes over time in light of this new pharmacy regulation on dispensing.

This study is limited to describing antibiotic availability in corner stores in the areas studied and does not represent Guatemala as a whole. Because no additional information was collected about the characteristics of the stores or store owners and medication suppliers, no causal relationships regarding study areas and antibiotic availability can be made. Future studies may consider collecting sociodemographic data to better understand if causal relationships exist across study communities in terms of the differences in the availability of antibiotics in different regions of Guatemala.

## Supporting information

S1 File Questionnaire Spanish/English. (PDF)

S1 Dataset Dataset availability of antibiotics in corner stores of Guatemala. (XLSX)
