# Supplementary material for: Availability of over-the-counter antibiotics in Guatemalan corner stores
Source: PLoS One. 2020 Sep 25;15(9):e0239873. doi: 10.1371/journal.pone.0239873 (PMC7518585; doi:10.1371/journal.pone.0239873)
Supplement: S1 File — (PDF) [file pone.0239873.s001.pdf]

Cuestionario para tiendas

1. Código: XX-TI-YY
   XX: código de comunidad
   TI: referencia a tienda
   YY: número correlativo

2. Punto GPS:

3. Dirección:

4. Hola, mi nombre es _____, vengo de la Universidad del Valle. Estamos haciendo un pequeño estudio para documentar el tipo de antibioticos que hay disponibles en esta comunidad. ¿Podemos hablar con el encargado de la tienda?
   a. Sí.
   b. No. Fin del cuestionario.

5. Hola, mi nombre es _____. Trabajamos en la Universidad del Valle de Guatemala y estamos haciendo un estudio para describir los tipos de antibioticos que se venden en esta comunidad. Este estudio nos ayudará a comprender qué medicamentos están disponibles para los residentes de esta comunidad. Esta encuesta tomará aproximadamente 5 minutos. La participacion es completamente voluntaria. Esto significa que usted no tiene que participar si no lo desea. Si está de acuerdo en participar, anotaremos los tipos de medicamentos que venden usando un cuestionario. No registraremos su nombre ni el nombre del establecimiento. Mantendremos en privado toda la informacion que usted nos de y no la compartiremos con personas afuera del estudio. Existe un riesgo minimo de perder la confidencialidad de la informacion que usted nos de. Sin embargo, haremos todo lo posible para que esto no sucede, pero si sucede, anticipamos que no tendra repercusiones negativas, dado que no vamos a registrar el nombre del establecimiento ni el suyo. ¿Estaría dispuesto a participar?
   a. Sí.
   b. No. Fin del cuestionario

6. ¿Usted vende antibióticos? [Si surge alguna duda, aclarar con ejemplos, como tetraciclina, santemicina o amoxicilina]
   a. Sí.
   b. No. Fin de la participación.

7. Número de antibióticos diferentes que venden en la tienda:
   [Número]

8. Para cada antibiótico:
   a. Nombre comercial:________

   b. Nombre del principio activo:
      i. Amoxicilina
      ii. Ampicilina
      iii. Tetraciclina
      iv. Ciprofloxacina
      v. Sulfametoxazol/Trimetoprim
      vi. Otro :______

   c. Laboratorio:______

d. Forma farmaceutica:
    i. Tableta
    ii. Capsula
    iii. Suspensión
    iv. Liquido
    v. Polvo para suspensión
    vi. Inyeccion
    vii. Otro

e. Indique el la dosis (ej. número de miligramos en la tableta/capsula/polvo):
   [Número de miligramos]

f. Precio por unidad (precio por una dosis: ej. Una cápsula, una tableta):
   [Precio por unidad en quetzales]

g. Fotografia (opcional):

REPITE LA PREGUNTA 8 PARA CADA ANTIBIÓTICO DISPONIBLE EN LA TIENDA

Questionnaire for corner stores

1. Code: XX-TI-YY
   XX: community code
   TI: reference for store
   YY: consecutive number

2. GPS point:

3. Address:

4. Hello, my name is _____, I work at the Universidad del Valle. We are doing a short-study to document what kind of antibiotics available in this community. Can we speak with the person in charge of the tienda that is here today?
   a. Yes.
   b. No. End of questionnaire.

5. Hello, my name is _____. We work at the Universidad del Valle de Guatemala and we are doing a study to describe the types of antibiotics that are sold in this community. This study will help us understand what medications are available to residents of this community. This survey will take approximately 5 minutes. Participation is completely voluntary. This means that you don't have to participate if you don't want to. If you agree to participate, we will record the types of drugs you sell using a questionnaire. We will not record your name or the name of the establishment. We will keep all the information you give us private and will not share it with people outside the study. There is minimal risk of losing the confidentiality of the information you give us. However, we will do everything possible so that this does not happen, but if it does happen, we anticipate that it will not have negative repercussions, since we will not register the name of the establishment or yours. Would you be willing to participate?
   a. Yes.
   b. No. End of questionnaire

6. Do you sell antibiotics here? [If any doubts arise, clarify with examples, such as tetracycline, santemycin or amoxicillin]
   a. Yes.
   b. No. End of participation.

7. Number of different antibiotics sold in the corner store:
   [Number]

8. For each antibiotic:
   a. Brand name: ________
   b. Name of the active ingredient:
      i. Amoxicillin
      ii. Ampicillin
      iii. Tetracycline
      iv. Ciprofloxacin
      v. Sulfamethoxazole / Trimethoprim
      vi. Other: ______

   c. Laboratory: ______
   d. Pharmaceutical form:
      i. Tablet
      ii. Capsule

iii. Suspension
iv. Liquid
v. Powder for suspension
vi. Injection
vii. Other

e. Indicate the dose (eg. number of milligrams in the tablet / capsule / powder):
[Number of milligrams]

f. Price per unit (price for one dose: eg. one capsule, one tablet):
[Price per unit in quetzales]

g. Picture (optional):

REPEAT QUESTION 8 FOR EVERY ANTIBIOTIC AVAILABLE IN THE CORNER STORE
